# Comparison of blood and lymph node cells after intramuscular injection with HIV envelope immunogens

**DOI:** 10.3389/fimmu.2022.991509

**Published:** 2022-10-05

**Authors:** Suzanne Day, Charandeep Kaur, Hannah M. Cheeseman, Emily de Groot, Leon R. McFarlane, Maniola Tanaka, Sofia Coelho, Tom Cole, Nana-Marie Lemm, Adrian Lim, Rogier W. Sanders, Becca Asquith, Robin J. Shattock, Katrina M. Pollock

**Affiliations:** ^1^ Department of Infectious Disease, Imperial College London, London, United Kingdom; ^2^ National Institute for Health and Care Research (NIHR) Imperial Clinical Research Facility, Imperial College Healthcare National Health Service (NHS) Trust, London, United Kingdom; ^3^ Department of Breast Radiology, Charing Cross Hospital, Imperial College Healthcare National Health Service (NHS) Trust, London, United Kingdom; ^4^ Medical Microbiology and Infection Prevention, Amsterdam University Medical Centers (UMC), University of Amsterdam, Amsterdam, Netherlands; ^5^ Infectious Diseases, Amsterdam Institute for Infection and Immunity, Amsterdam, Netherlands; ^6^ Dept Microbiology and Immunology, Weill Cornell Medical Center, Cornell University, New York, NY, United States

**Keywords:** lymph node cells, vaccine, HIV, T follicular helper cell, T cell, HIV envelope, CD4+ T cell, envelope

## Abstract

**Background:**

Harnessing CD4+ T cell help in the lymph nodes through rational antigen design could enhance formation of broadly neutralizing antibodies (bNAbs) during experimental HIV immunization. This process has remained hidden due to difficulty with direct study, with clinical studies instead focusing on responses in the blood as a proxy for the secondary lymphoid tissue.

**Methods:**

To address this, lymph node cells (LNC) were collected using ultrasound guided fine needle aspiration of axillary lymph nodes from 11 HIV negative participants in an experimental HIV immunogen study (European AIDS Vaccine Initiative EAVI2020_01 study, NCT04046978). Cells from lymph node and blood (PBMC), were collected after intramuscular injection with HIV Env Mosaic immunogens based on HIV Envelope glycoprotein and combined with a liposomal toll-like receptor-4 adjuvant; monophosphoryl lipid A. Simultaneously sampled cells from both blood and lymph node in the same donors were compared for phenotype, function, and antigen-specificity.

**Results:**

Unsupervised cluster analysis revealed tissue-specific differences in abundance, distribution, and functional response of LNC compared with PBMC. Monocytes were virtually absent from LNC, which were significantly enriched for CD4+ T cells compared with CD8+ T cells. T follicular helper cells with germinal center features were enriched in LNC, which contained specific CD4+ and CD8+ T cell subsets including CD4+ T cells that responded after a single injection with HIV Env Mosaic immunogens combined with adjuvant. Tissue-specific differences in response to an MHC-II dependent superantigen, staphylococcal enterotoxin B, indicated divergence in antigen presentation function between blood and lymph node.

**Conclusions:**

LNC are phenotypically and functionally distinct from PBMC, suggesting that whole blood is only a limited proxy of the T cell lymphatic response to immunization. HIV-specific CD4+ T cells in the lymph node are rapidly inducible upon experimental injection with HIV immunogens. Monitoring evolution of CD4+ T cell memory in LNC with repeated experimental HIV immunization could indicate the strategies most likely to be successful in inducing HIV-specific bNAbs.

## Introduction

Vaccine strategies to induce broadly neutralizing antibodies against HIV involve repeated exposure to conserved immunodominant epitopes to mimic the process in natural infection. Immunization strategies that enhance persistent antigen exposure are used. This approach increased frequencies of total and HIV-specific Tfh in the germinal center (GC) and autologous tier 2 neutralizing antibody responses in non-human primates (NHP) (1). Presently, clinical vaccine research relies on measurements using the blood as proxy, leading to a diffuse and potentially inaccurate picture with respect to the cell subtypes, magnitude, and kinetics of the lymph node response. The mechanism of this reaction has remained hidden because of challenges in the direct study of human lymph nodes. CD4+ T cell help in the GC determines B cell clonal survival and proliferation during the response to protein antigen. This process determines the induction of B cells capable of producing broadly neutralizing antibodies (bNAbs). The quality of Env-specific Tfh in the lymph node and expression of Tfh-genes in Env-specific CD4+ T cells in the peripheral blood was critical in the formation of neutralizing antibody in response to vaccination with adjuvanted HIV envelope trimer in NHP (2). There are limited data from *in vivo* lymphatic responses after vaccination in humans, reviewed in (3). Responses to tuberculin purified protein derivative have been observed in lymph nodes two days after an intradermal injection of tuberculin (4). In children immunized with a quadrivalent influenza vaccine, CD4+ Tfh accumulated in tonsils and were associated with the frequency of influenza strain specific antibody secreting cells (5). In people living with HIV on antiretroviral therapy, Tfh in lymph nodes were relatively enriched and the frequencies dropped after influenza vaccination, a pattern not seen in HIV negative volunteers (6). Tracking human lymphatic tissue responding to repeated intramuscular injection would iteratively inform the ability of rationally designed HIV immunogens to determine this cellular evolution. Systems vaccinology would be fundamentally enhanced by data from the direct *in vivo* study of reacting human secondary lymphoid tissue (7). Together, this would accelerate the process of HIV vaccine discovery.

The process by which pathogen-activated B cells undergo somatic hypermutation, positive selection and differentiation in the GC is regulated by specialist CD4+ T follicular helper cells, *via* co-stimulatory molecules and cytokines (8). Studies of B cell evolution in GC reactions following COVID-19 vaccination indicate fundamental differences in the B cell response in blood and lymph node, with blood plasmablasts accumulating antigen-specific somatic hypermutation at a lower rate than GC B cells (9). HIV vaccine design employing the Envelope (Env) glycoprotein that promoted effective GC Tfh help would be expected to stimulate a robust GC B cell response facilitating the development of bNAbs.

Our ability to study this CD4+ T cell help is limited by access to the tissues where the different subtypes reside; secondary lymphoid tissue including lymph nodes, thoracic duct, and blood. Activation of the related cells in the blood, circulating Tfh (cTfh) occurs after vaccination and correlates with the serological response (10, 11). In the thoracic duct, Tfh-like cells have overlapping phenotypic and transcriptional features between lymph node and blood (12). In the lymph node, the functional relationship between CD4+CXCR5+ GC Tfh, which highly express programmed death 1 (PD-1), and their circulating counterparts remains an outstanding question in the field.

The biological parameters governing human lymph node reactions are of particular interest for the development of HIV vaccine designs because of the requirement to induce bNAbs. These are antibodies with extraordinary features that deliver breadth and potency of neutralization (13). Direct observation of the responding GC Tfh would provide fundamental insight into the CD4+ T helper cell response generated by this strategic immunization approach. In addition to CD4+ GC Tfh, other tissue resident or tissue associated T cells including CD8+ T cells may be activated in the lymph node by immunization. Data on many of these cell subtypes during clinical studies of novel vaccines have not previously been reported. The direct study of these forms of T cell memory induced in secondary lymphoid tissue could enhance vaccine design.

The EAVI2020_01 HIV immunogen study is a clinical study at Imperial College London, UK. The study, (European AIDS Vaccine Initiative EAVI2020_01 study, NCT04046978), employs repeated intramuscular injection of rationally designed HIV Env Mosaic immunogens (HIV Mosaic). These polypeptide proteins are based on the Env glycoprotein stabilized in the native conformation and have been specifically designed to induce broadly neutralizing antibodies against HIV (14–17). Ultrasound (US) guided fine needle aspiration (FNA) of the lymph node is a well-tolerated diagnostic technique that is not yet embedded within clinical vaccine research but has nevertheless proven feasible in a research setting (18). In the EAVI2020_01 study, volunteers were offered the opportunity to donate lymph node cells (LNC) using this technique at sampling study visits. Here we demonstrate the *in vivo* study of critical T cell types that reside in secondary lymphoid tissue contralateral and ipsilateral to the site of injection, which are phenotypically and functionally distinct from their circulating counterparts.

## Materials and methods

### Study design

The lymph node study design was an experimental medicine study involving healthy HIV negative volunteers enrolled into the main EAVI2020_01 study. In the main study, which is on-going, participants were enrolled and received intramuscular injections of the experimental HIV immunogen with liposomal MPLA into the deltoid muscle of their choice. Participants were followed up at routine visits to donate blood for immunogenicity analysis. Participants were invited to take part in the lymph node study and attended an additional visit after injection of the first or second immunogen. The EAVI2020_01 study (NCT04046978) was approved by London – Fulham Research Ethics Committee (18/LO/2196). All volunteers provided written informed consent to participate in both the main study and lymph node study.

### FNA sampling schedule

**Figure 1:**
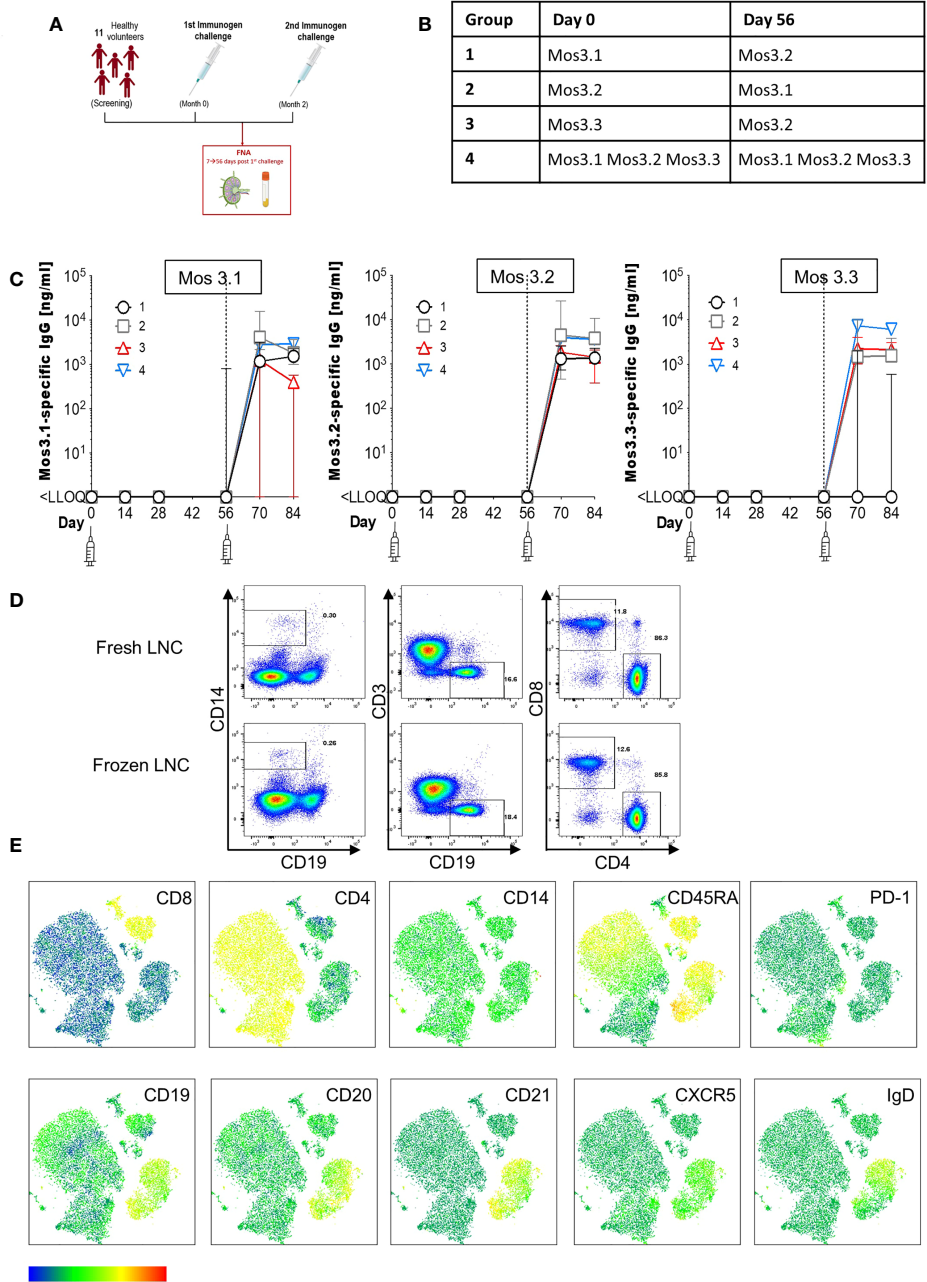
Induction of HIV-specific antibodies and lymph node cell yields after injection with HIV Mosaic immunogens. **(A)** Study overview: 11 participants chose to join the lymph node study and proceeded to sampling. All participants except one (who had the FNA after the second injection) had the lymph node FNA after the first study injection. **(B)** The schedule of injections for the EAVI2020_01 study for groups 1 to 4 is shown where participants were challenged with a single HIV Mosaic immunogen (Mos 3.1, 3.2 or 3.3) at a dose level of 100 µg or a cocktail of all three (group 4) at 33 µg coupled with a liposomal MPLA adjuvant. **(C)** Detection of HIV-specific IgG in serum of individuals after receiving two injections with HIV immunogens. Responses at six timepoints during the study are shown in the 11 participants who underwent FNA. Graphs show IgG in ng/mL against Mos 3.1 (left panel), Mos 3.2 (middle panel) and Mos3.3 (right panel) at baseline, between prime and boost doses, and after the boost dose at Day 56. Responses from participants in each group are shown with group 1 black circles (n=3), group 2 grey squares (n=3), group 3 red triangles (n=3) and group 4 blue inverted triangles (n=2). **(D)** Comparison of fresh and frozen lymph node cell composition: LNC from one participant were either stained at once (top row), or viably cryopreserved, thawed and then stained (bottom row) with a cocktail of 15 cell surface phenotyping markers before fixing with fixation buffer. Events were acquired on a BD Fortessa flow cytometer. Events were gated according to immunophenotyping to define major cellular subsets in LNC for CD14+CD19- monocytes (left panel), CD19+CD3- B cells (middle panel) and CD4+ and CD8+ T cell populations (right panel). Up-stream gating of cells was on the lymphocyte population with doublet (FSC-A/W, SSC-A/W) and dead cell exclusion (not shown). **(E)** FlowSOM clusters were obtained based on typical phenotyping markers. Expression of CD8, CD4, CD14, CD45RA, PD-1, CD19, CD20, CD21, CXCR5 and IgD is shown on each FlowSOM cluster for frozen LNC imposed on a t-SNE representation. The scale of expression is shown in the heatmap legend from low (blue) to high (red). Cell types enriched for expression of each marker are visible in the light green to orange color range. Clusters were derived from events acquired from a fine needle aspirate of frozen LNC as described above.

Volunteers underwent sampling of axillary lymph nodes following a physical examination to determine the most appropriate nodes for sampling. Except for one participant, sampling in the study occurred between the first and second immunogen injections in participants from Groups 1 to 4 of the EAVI2020_01 HIV Mosaic study (**Figure 1A**). Paired peripheral whole blood samples (42 mL) were taken contemporaneously.

### Study immunogens

Participants had received one or all of three rationally designed Mosaic HIV envelope glycoprotein immunogens Mos3.1, Mos3.2 or Mos3.3 (**Figure 1B**). These model immunogens mimic the native HIV-1 viral trimers consisting of gp120 and g41 and have been modified to form the soluble molecule gp140. Mosaic immunogens were designed using algorithms aimed at eliciting responses against conserved HIV neutralization epitopes. Model immunogens were manufactured by Polymun to cGMP standards. The immunogens were given with an adjuvant; the liposomal form of the TLR-4 agonist MPLA also manufactured by Polymun.

### Schedule of injections

During the study, the EAVI2020_01 immunogens were administered into the deltoid muscle of the upper arm (**Table 1**). Arm selection, (left or right) was according to participant preference. The following immunogens were used Mos 3.1 100 μg, Mos 3.2 100 μg, Mos 3.3 100 μg each with 500 μg of a liposomal form of the adjuvant monophosphoryl lipid A (MPLA), MPLA-5 adjuvant. Where a cocktail of three Mos immunogens were used, 33 μg of each was injected.

**Table 1:** Participant demographics.

Participant number	Age	Sex	Ethnicity	Site of study injection	Time of FNA after study injection (days)
70	20	F	White British	left deltoid	41
79	38	M	White British	left deltoid	35
69	27	F	Any other white background	right deltoid	34
52	31	M	Black African	left deltoid	50
82	46	F	White British	left deltoid	42
71	32	M	Any other white background	right deltoid	36
75	26	M	White British	left deltoid	34
76	44	M	Any other white background	right deltoid	14
80	25	F	White British	right deltoid	44
63*	43	M	White British	left deltoid	36
59	24	F	Any other white background	left deltoid	16

*63 had the FNA after the second injection into the left deltoid, all others had the FNA after the first.

### Antigen-Specific IgG ELISA

Antigen specific IgG antibodies were measured in sera using in-house standardized conventional ELISA platforms. In brief, 96-well high-binding plates (Greiner, Kremsmünster, Austria) were coated with anti-human kappa and lambda light chain specific mouse antibodies (Southern Biotech, Birmingham, AL) at 1:1 ratio diluted 1:500 in PBS or antigen (1 µg/mL of either Mosaic 3.1, Mosaic 3.2, or Mosaic 3.3 protein (Polymun, Austria) for 1 hour at 37°C. After blocking with block buffer (5% BSA (Sigma-Aldrich, St. Louis, MO), 0·05% Tween 20 (Fisher, Pittsburgh, PA) in D-PBS (Sigma-Aldrich)) samples were initially screened at 1:50 dilution (then titrated to optimal dilutions). Serial dilutions (1:5) of immunoglobulin standards (purified human IgG starting at 1 µg/mL) were added in triplicate to kappa/lambda capture antibody-coated wells and incubated for 1 hour at 37°C. Secondary antibody, HRP-conjugated anti-human IgG (Sigma-Aldrich, St. Louis, MO), was added at 1:20,000 dilution and incubated for 1 hour at 37°C. Plates were developed with SureBlue TMB substrate (KPL, Insight Biotechnology, London, UK). The reaction was stopped after 5 minutes by adding TMB stop solution (KPL, Insight Biotechnology) and the absorbance read at 450 nm on a VersaMax 96 well microplate reader (Molecular Devices, Sunnyvale, CA). The ELISA data were expressed as positive if the blank-subtracted OD 450 nm was above the pre-determined cut-off of OD 0·2 nm and values were on the linear range of the curve. To ensure assay sensitivity, a positive control composed of positive pooled plasma samples was used. Analyses of the data were performed using SoftMax Pro GxP software (version 6.5, Molecular Devices, Sunnyvale, CA).

### Cellular subset phenotyping

Briefly, LNC and PBMC were stained in FACS buffer using a pre-optimized cocktail of fluorochrome conjugated antibodies to identify different immune cell subtypes by means of flow cytometry. The cocktail was designed for the identification of CD14+ monocytes, CD4 and CD8+ T cell memory subsets, B cell subsets and Tfh and cTfh. Cells were resuspended in fixation buffer and events acquired within 18 hours on a Becton Dickinson Fortessa LSR-SORP equipped with 20 mW 355 nm, 50 mW 405 nm, 50 mW 488 nm, 50 mW 561 nm, 20 mW 633 nm lasers and a ND1.0 filter in front of the FSC photodiode. Acquisition was set to record live CD3+ lymphocytes after dead cell and doublet exclusion (FSC-A/W, SSC-A/W gating). Data were reported as % of parent population.

### Activation induced marker assay

This was performed on PBMC and LNC isolated from participants post-injection. All antibodies were pre-titrated to optimal dilutions. Cryopreserved PBMC and LNC were thawed and rested at 5x10^6^ cells/mL for 3 hours in 10% human assay buffer (10% HAB; 10% human serum (Sigma-Aldrich, St. Louis, MO) in RPMI media) at 37°C, 5% CO_2_. After resting, 0.5 µg/mL CD40 blocking antibody (Miltenyi Biotech, Bergisch Gladbach, Germany) and CXCR5-BB515 (Clone RF8B2; BD Biosciences, San Diego, CA) were added for a 15-minute incubation at 37°C. Cells were then stimulated for 18 hours at 37°C with various stimulation conditions - media only, EAVI Mosaic peptide pool (0.5 µg/mL) and SEB (staphylococcal enterotoxin B; Sigma-Aldrich, St. Louis, MO; at 1µg/mL). After antigen stimulation, cells were stained with fixable viability dye eFluor506 (eBioscience, San Diego, CA), CD3 BUV395 (UCHT1), CD4 BUV496 (SK3), CD8 V500 (RPA-T8), CD14 V500 (M5E2), CD19 V500 (H1B19), CD154 PE (TRAP-1; all BD Biosciences, San Diego, CA), CD45RA PE-Dazzle (HI100), PD-L1 PE-Cy7 (29E.2AE), OX40 APC (ACT35), CD25 APC-Fire750 (BC96), CD69 BV650 (FN50) and PD-1 BV421 (EH12.2H7; all BioLegend, San Diego, CA). Cells were resuspended in Fixation buffer (eBioscience) and permeabilized before intracellular staining for IRF-4 PerCP Cy5.5 (IRF4.3E4; BioLegend) prior to flow cytometry analysis measured on a Becton Dickinson FortessaLSR-SORP equipped with 20 mW 355 nm, 50 mW 405 nm, 50 mW 488 nm, 50 mW 561 nm, 20 mW 633 nm lasers and a ND1.0 filter in front of the FSC photodiode. Acquisition was set to record 50,000 live CD3+ lymphocytes after dead cell and doublet exclusion (FSC-A/W, SSC-A/W gating). Data were reported as % of parent population, with negative control subtracted from peptide and positive control stimulated data.

### Interferon regulatory factor 4 assay

Cryopreserved cells from LNC and PBMC were thawed and rested for 3 hours at a concentration of 1x10^7^/mL in 10% HAB (10% human serum (Sigma-Aldrich, St. Louis, MO) in RPMI media) at 37°C, 5% CO2. After resting, CXCR5-BB515 (Clone RF8B2; BD Biosciences, San Diego, CA) was added for a 15-minute incubation at 37°C. Cells were then stimulated with R10 media only (negative control), EAVI Mosaic peptide pool (0.5 µg/mL) or SEB (staphylococcal enterotoxin B; 1 µg/mL) as a positive control, for 18 hours at 37°C, 5% CO2. After antigen stimulation, cells were stained with NIR viability dye, CD4 BUV496 (SK3), CD8 BUV395 (RPA-T8; both BD Biosciences), CD3 BV785 (SK7), CD14 APC-Cy7 (M5E2), CD19 APC-Cy7 (SJ25C1), CD45RA PerCPCy5.5 (HI100), CD28 BV605 (CD28.2), CCR7 PE-Cy7 (G043H7), CD95 AlexaFluor647 (DX2), PD-1 BV421 (EH12.2H7), CXCR5 BB515 (RF8B2), CD69 BV650 (FN50), CD154 PE (clone 24–31) and CD137 BV711 (4B4-1; all BioLegend, San Diego, CA). Cells were resuspended in Fixation buffer (eBioscience) and permeabilized before intracellular staining for IRF-4 PE-CF594 (IRF4.3E4; BD Biosciences) prior to flow cytometry analysis measured on a Becton Dickinson FortessaLSR-SORP equipped with 20 mW 355 nm, 50 mW 405 nm, 50 mW 488 nm, 50 mW 561 nm, 20 mW 633 nm lasers and a ND1.0 filter in front of the FSC photodiode. Acquisition was set to record 50,000 live CD3+ lymphocytes after dead cell and doublet exclusion (FSC-A/W, SSC-A/W gating). Data were reported as % of parent population, with negative control subtracted from peptide and positive control stimulated data.

### Flow cytometry data analysis

Analysis was performed using FlowJo software v10.6 (Treestar, Ashland, OR). Sequential data analysis was conducted according to standard immunophenotyping protocols. Unsupervised analysis was conducted using the t-SNE Plugin. Data were cleaned by gating on live singlets before concatenation of the combined files. The subsequent t-SNE was conducted using FlowJo with 1000 iterations and perplexity of 30.

### Statistical analysis

Data were assumed to be non-normally distributed given the small sample size. Continuous variables were summarized by means of median and interquartile range (IQR) and were compared using a two-sided Mann-Whitney U test using GraphPad Prism V8/9. A p value of <0.05 was significant.

## Results

### Lymph node cell yields and induction of HIV-specific antibodies

To determine the feasibility, safety, and tolerability of US guided FNA of the ipsilateral and contralateral axillae after HIV Mosaic immunogen injection into the deltoid muscle, participants in the EAVI2020_01 study were offered the opportunity to donate LNC with paired phlebotomy. Twelve participants took part in the lymph node study between 10^th^ June 2021 to 5^th^ August 2021. One participant had a visit scheduled outside the allotted time window between immunogens and could not proceed. Eleven participants proceeded to axillary lymph node FNA according to the study design and received two injections of HIV Mosaic immunogens at Day 0 and Day 56 **(** **Figure 1A**). All immunogens were given at a dose of 100 µg except for the cocktail of three immunogens where the dose was 33 µg per immunogen. Each injection was given together with liposomal Monophosphoryl Lipid A (MPLA) at a dose of 500 µg. Each group; 1-4, received a different HIV Mosaic immunogen, Mos 3.1, Mos 3.2 and/or Mos 3.3 or combination of immunogens at Day 0 and Day 56 (**Figure 1B**).

Participants were between 20 and 46 years of age, with a median age of 31 years. All participants had tested negative in a fourth generation HIV test upon entry into the EAVI2020_01 study. Of the participants, 5/11 (45%) were female and 6/11 (55%) were male. The majority, 55% (6/11), reported their ethnicity as ‘White British’, 4/11 (36%) reported their ethnicity as ‘Any other white background’ and 1/11 (9%) as ‘Black African’ (**Table 1**).

To determine whether HIV-specific responses were raised against each of the HIV Mosaic immunogens, Mos 3.1, Mos 3.2, and Mos 3.3-specific IgG was measured by ELISA from sera at six timepoints during the FNA study. HIV-specific antibody responses were not detectable at baseline in any of the 11 participants confirming antigen-naïve status. HIV-specific binding IgG was not detectable against any of these proteins prior to the boost dose of immunogen at Day 56. Responses against Mos3.2 were detectable in all participants 14 days after the booster dose in sera from all participants. 1/3 participants had received the Group 3 schedule of immunogens, where no Mos3.1 was given, and did not raise a detectable IgG response against Mos3.1. 2/3 participants had received the Group 1 schedule of immunogens, where no Mos3.3 was given, and did not raise a detectable IgG response against Mos3.3 (**Figure 1C** and **Figure S1**).

LNC from lymph nodes in the axillae that were ipsilateral and contralateral to the site of injection were collected from the n=11 individuals; median (IQR) 1.15 (0.33 – 4.34) million cells with viability 90.6% (59.3%-96.9%) prior to cryopreservation (**Table S1**). There was no change in lymphocyte cell subset composition before and after cryopreservation for PBMC or LNC (**Figure 1D** and **Figure S1**). Immunophenotyping markers were clearly expressed on cryopreserved LNC using unsupervised clustering analysis allowing definition of the three major lymphocyte cell types, CD4+ T cells, CD8+ T cells and B cells, and distinction of cell subtypes by expression of CD8, CD4, CD14, CD45RA, PD-1, CD19, CD20, CD21, CXCR5 and IgD (**Figure 1E**).

The FNA procedure was well tolerated without serious or high-grade adverse events. Adverse events were assessed 15-30 minutes post FNA and at the follow up visit. There were no serious complications or safety concerns, and the procedure was well tolerated. All events were mild, there were no adverse events that were moderate or severe, and no serious adverse events related to the procedure. Altogether 10/11 (91%) individuals reported adverse events immediately, or at the follow-up visit. Post-procedure, 7/11 (64%) participants reported adverse events and 4/11 (36%) reported no adverse events. The most common immediate event was mild tenderness/pain at the FNA site 6/11 (55%), followed by small bruising at the FNA site 2/11 (18%). One participant experienced superficial mild swelling of the FNA site that resolved by the next day. One participant had slight paraesthesia at the FNA site which resolved. One participant became nauseous post FNA, with no other associated symptoms and a normal clinical examination. This resolved and they were able to have FNA of their contralateral axilla without any further problems. One participant reported bruising the day after the procedure. Most adverse events reported at follow-up 6/10 (60%) resolved within 48 hours and all resolved within five days.

### The cellular landscape of blood and lymph node is divergent

**Figure 2:**
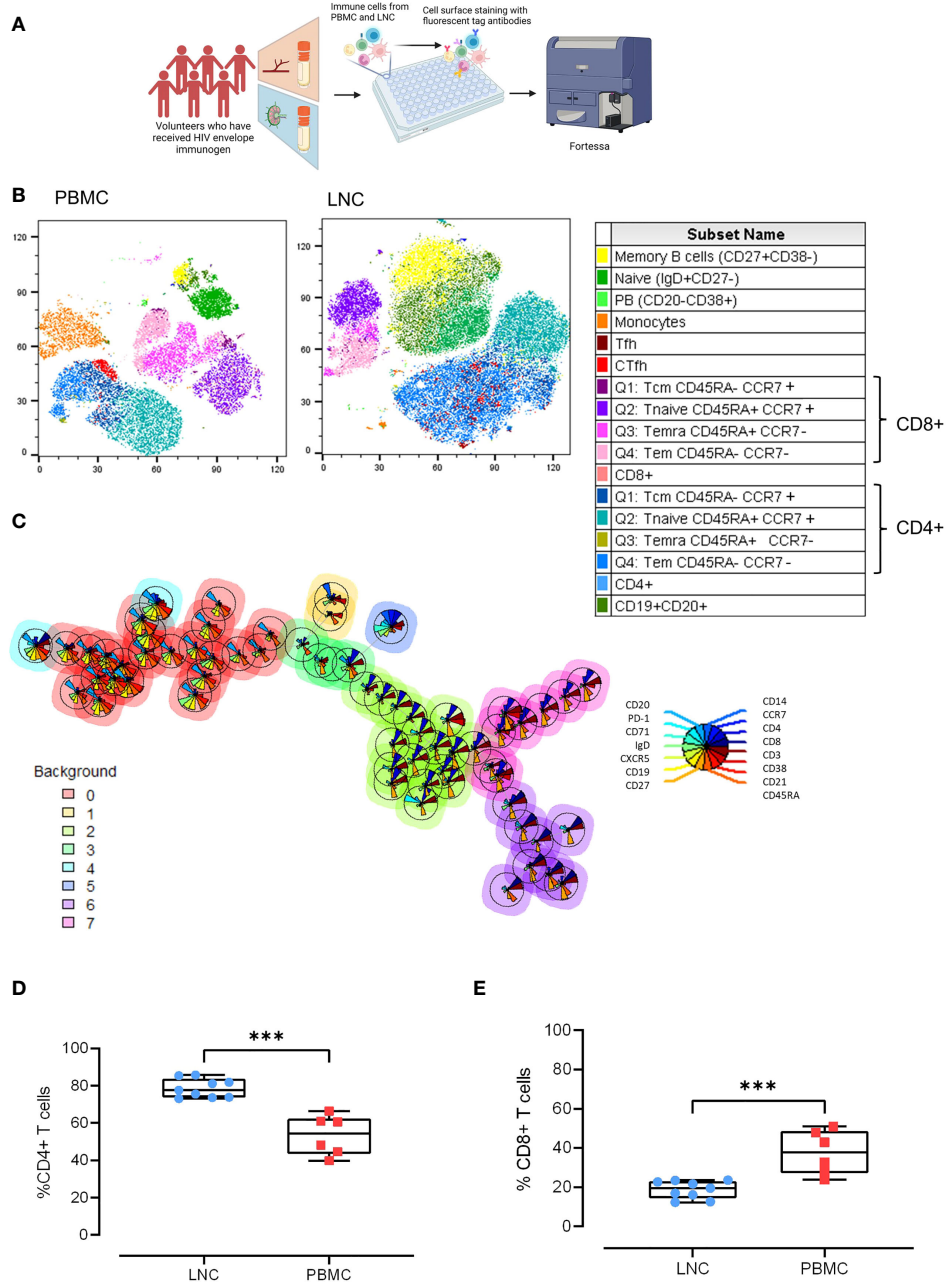
Comparison of the cellular landscape in blood and lymph node. **(A)** Paired PBMC and LNC from six participants were stained with a cocktail of 15 cell surface phenotyping markers and events acquired on a BD Fortessa flow cytometer. **(B)** t-distributed Stochastic Neighbor Embedding (t-SNE) representation of peripheral blood mononuclear cells (left panel) and lymph node cells (middle panel). The data were manually cleaned (gated on FlowJo on time> Lymphocytes> FCS singlets> SSC> Singlets> Viability) and concatenated on the down sample of each participant’s live population- 10,000 events. Seventeen cell subsets were defined by manual cell gating and are displayed on the plots by color distribution as shown in the legend (right panel). 1. Memory B cells: CD19+ CD20+ CD27+ CD38-; 2. Naïve B cells: CD19+ CD20+ IgD+ CD27-; 3. Plasmablasts: CD19+CD20-CD38+; 4. Monocytes: CD14+CD19-; 5. T follicular helper cells (Tfh):CD4+CD19-CD45RA-CXCR5+PD-1+; 6. Circulating T follicular helper cells (cTfh): CD4+CD19-CD45RA-CXCR5+; 7. CD8+T central memory cells (Tcm) CD45RA+ CCR7+; 8. CD8+Tnaive cells CD45RA+CCR7+; 9. CD8+Temra cells CD45RA+ CCR7-; 10. CD8+T effector memory cells (Tem) CD45RA- CCR7-; 11. CD8+ T cells; 12. CD4+T central memory cells (Tcm): CD45RA- CCR7+; 13. CD4+Tnaive cells: CD45RA+CCR7+; 14. CD4+Temra cells: CD45RA+ CCR7-; 15. CD4+T effector memory cells: (Tem) CD45RA- CCR7-; 16. CD4+ T cells; 17. B cells CD19+CD20+. **(C)** Self-organizing maps (SOM) of the data represented in minimal spanning trees meta-clustered using FlowSOM from concatenated data from LNC from n=6 participants. The background color of each node represents one of eight metaclusters from 0 to 7 (legend left). The individual star charts for each node show the median intensities of each phenotypic marker (legend right). **(D, E)** Paired PBMC and LNC from n=6 participants’ data were cleaned and gated according to the gating strategy. Each panel shows the individual data points for LNC (blue circles), PBMC (red squares) and the median and interquartile range as a box and whisker plot. Frequency of CD4+ T cells in live CD3+ T cells **(D)**, and frequency of CD8+ T cells in live CD3+ T cells **(E)**. The results of Mann-Whitney U tests are shown with ***p<0.001.

To probe the cellular composition of the different human tissues; blood, and lymph node, paired LNC and PBMC from six participants were stained with an optimized cocktail of 15 fluorochrome-conjugated antibodies defining the major lymphocyte and monocyte lineages and cell subsets (**Figure 2A**). Unsupervised clustering analysis of the concatenated and down sampled immunophenotyping data generated seventeen cell clusters representing monocytes, and T and B cell memory subsets. The breadth and frequency of cell-types were markedly different between blood and lymph node. CD14+ monocytes, enriched in PBMC, were virtually absent in LNC. B and CD4+ T cells were dominant in LNC with CD8+ T cells forming a smaller cluster. Naive and memory CD4+ and CD8+ T cells were present in both tissues. Memory and naïve B cells were abundant in LNC. In PBMC, cTfh were strongly clustered. In LNC, cTfh and Tfh were distributed in the CD4+ T cell cluster (**Figure 2B**). Self-organizing maps (SOM) of the data were automatically meta-clustered in a minimal spanning tree. There were three dominant meta clusters, hierarchically related to one another; CD19+CD20+ B cells (red meta cluster, 0), CD3+CD4+ T cells (light green meta cluster, 2) and CD3+ CD8+ T cells (purple meta cluster, 6) with two intermediate meta clusters (**Figure 2C**).

Findings from unsupervised analyses were tested in sequential two-dimensional analyses. The paucity of CD14+ monocytes in LNC was confirmed, median (IQR) 0.42% (0.28-0.7%) compared with PBMC, 11.1% (8.3%-12.1%), (p<0.001). In live CD3+ T cells, CD4+ T cells were significantly enriched in LNC compared with PBMC, median (IQR) 77.6% (73.8-83.7%) vs 54.5% (43.5-62.35%), (p<0.001) (**Figure 2D**). By contrast, frequencies of CD8+ T cells were higher in PBMC than in LNC, median (IQR) 37.9% (27.2-48.6%) vs.) 19.6% (14.4-23.1%), (p<0.001) (**Figure 2E**). The median (IQR) CD4:CD8 ratio of T cell subset frequency was significantly higher for LNC than PBMC median (IQR) 4.0 (3.2-5.9) vs. 1.5 (0.9-2.3), (p<0.001).

### Memory T and B cell subset distribution is tissue specific

**Figure 3:**
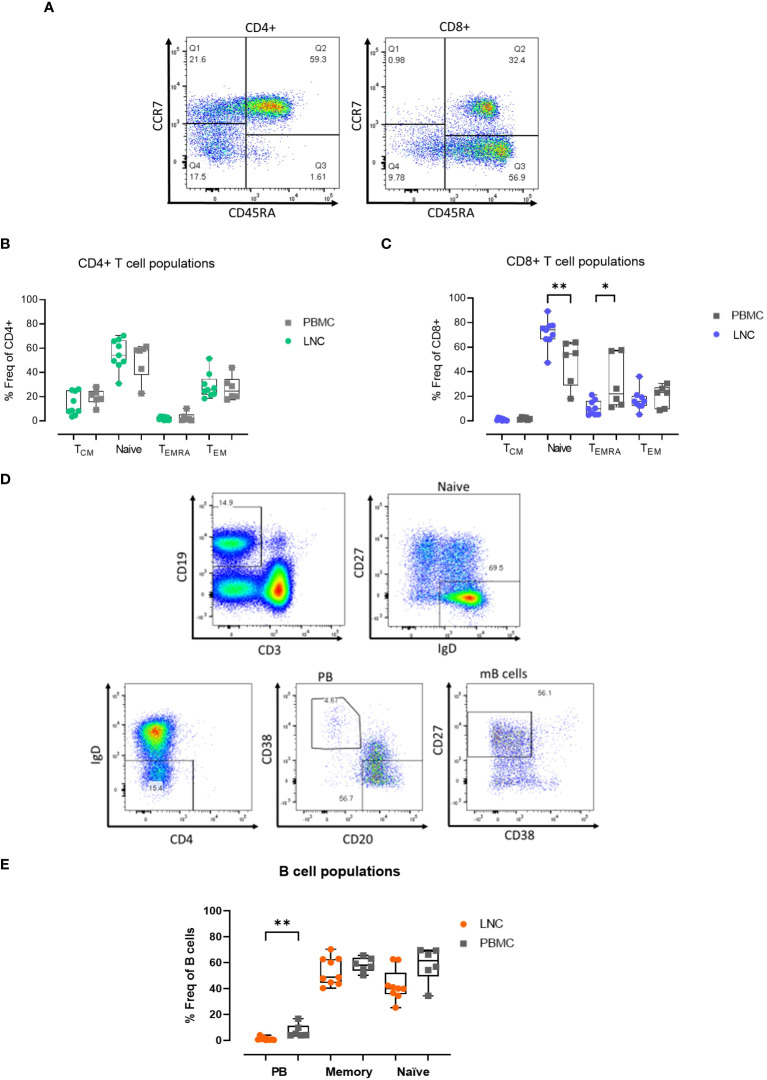
Memory T and B cell subset distribution in blood and lymph node. **(A)** Representative gating strategy for CD4+ and CD8+ T cell memory subset populations for surface expression of CCR7 and CD45RA on live, CD3+ CD4+/CD8+ singlets; defined as Tcm, CCR7+ CD45RA- (Q1), Naïve; CCR7+ CD45RA+ (Q2) Temra; CCR7- CD45RA+ (Q3) and Tem; CCR7- CD45RA- (Q4) subsets for CD4+ T cells (left panel) and CD8+ T cells (right panel). **(B)** Comparison of frequencies of CD4+ T cell memory populations in PBMC and LNC. **(C)** Comparison of frequencies of CD8+ T cell memory populations in PBMC and LNC. **(D)** Representative gating strategy of memory B cell subsets with CD19+CD3- live B cells gated for naive IgD+CD27-, IgD-CD38+CD20- plasmablasts and CD27+CD38- memory B cell subsets. **(E)** Comparison of frequency of B cell subsets in PBMC and LNC. The results of Mann-Whitney U tests are shown with *p<0.05, **p<0.01.

Given that immune challenge with HIV Mosaic immunogen may impact T cell memory in reacting lymph nodes, the composition of T cell memory subsets in LNC was examined. Memory subsets in T cells were defined as naïve; CCR7+CD45RA+, Tcm; CCR7+CD45RA-, Temra; CCR7- CD45RA+ and Tem; CCR7-CD45RA- (**Figure 3A**). In CD4+ T cells, the frequency of the memory subsets in blood and lymph node was similar (**Figure 3B**). Naive CD8+ T cells were enriched in LNC compared with PBMC, median (IQR) 73.9% (66.5-77.5%) vs 54.4% (28.8-63.1%), (p<0.01). In PBMC, CCR7-CD45RA+ (Temra) CD8+ T cells, were more abundant than in LNC, (p<0.05) (**Figure 3C**).

There was a trend toward a higher frequency of CD19+CD3- B cells in LNC than PBMC; median (IQR) 14.3% (9.3-21.7%) vs. 7.5% (5.2-12.1%) (data not shown), reflecting earlier findings on t-SNE analysis. In CD19+CD3- B cells, CD27-IgD+ naïve cells, IgD-CD38+CD20- plasmablasts, and IgD-CD27+CD38- memory B cells were analyzed (**Figure 3D**). Plasmablasts were more abundant in the B cells found in PBMC than in LNC, median (IQR) 4.6% (3.8-11.3%) vs. 0.7% (0.3-1.5%), (p<0.01), whereas memory and naïve B cells were found in both compartments, with a trend towards a lower frequency of naïve B cells in the LNC (**Figure 3E**).

### Tfh with a germinal center (GC)-like phenotype are exclusive to the lymph node

**Figure 4:**
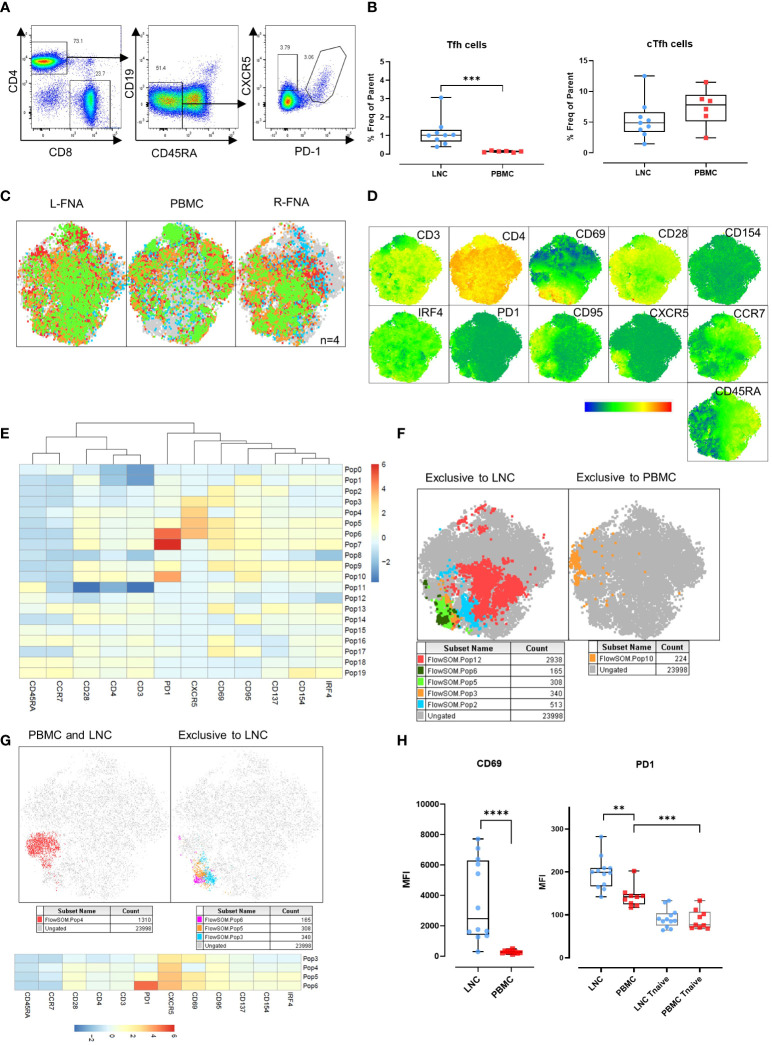
Germinal center (GC) Tfh in the lymph node, and circulating Tfh in blood and lymph node. **(A)** Representative gating strategy for T-follicular helper cells (Tfh) and circulating T-follicular helper cells (cTfh). Tfh were gated for live CD4+ T cells that were CD45RA-CD19- and expressing CXCR5 and PD-1. cTfh were gated for live CD4+ T cells expressing CXCR5 only. **(B)** Comparison of frequencies of Tfh (left panel) and cTfh (right panel) in CD4+ T cells in LNC and PBMC. Data are from n=6. The results of Mann-Whitney U tests are shown with *p<0.05, **p<0.01, ***p<0.001. **(C)** t-SNE from paired LNC and PBMC concatenated on live CD4+ T cells that were down sampled for 2063 events (CD4 T cells) from each participant (n=4). t-SNE was run with perplexity of 30. Data were gated to identify the samples for the left axilla fine needle aspirate (L-FNA), PBMC and the right axilla FNA (R-FNA), using SampleID (**Figure S2**). Color coding green, red, orange, and blue represents each of the different participants and grey dots represent the ungated cells that are absent in the subset. **(D)** Expression of immunophenotyping markers represented in heatmap statistical plots on tSNE generated by concatenating paired LNC and PBMC on live CD4+ T cells that were down sampled for 2063 events (CD4 T cells) from each participant (n=4), with the legend showing the scale of expression from low (in blue), to high (in red). **(E)** Population heatmap statistics for the twenty FlowSOM populations generated on tSNE of paired LNC and PBMC concatenated on live CD4+ T cells that were down sampled for 2063 events (CD4+ T cells) from each participant (n=4), showing the expression of the immunophenotyping and activation markers. **(F)** FlowSOM with 20 meta clusters applied on the concatenated CD4+ T cell data. From 20 different meta clusters, subpopulations exclusively occurring in LNC or PBMC were identified manually and are shown in the legend below each tSNE plot. **(G)** FlowSOM population overlay on t-SNE analysis of CXCR5+ CD4+ T cells found in PBMC and LNC (left panel) and in LNC exclusively (right panel). Subpopulations exclusively occurring in LNC or PBMC were identified manually and are shown in the legend below each tSNE plot **(H)** Comparison of the median fluorescence intensity (MFI) of CD69 and PD1 expression on CD4+ CXCR5+ CD45RA- T cells and CD45RA+CCR7+ naïve T cells expressing PD-1 in LNC and PBMC. Results show analysis by Mann-Whitney U tests **p<0.01, ***p<0.001, ****p<0.0001.

CD4+ Tfh provide help to B cells responding to T-dependent antigens through GC formation and induction of affinity matured plasma cells and memory B cells. Previously, the study of similar cells in the circulation, cTfh, as a proxy of canonical GC Tfh has predominated because of difficulty in direct study. However, the relationship between these cell types is not determined and neither is their relative importance in determining HIV specific B cell clonal selection. To establish the relationship between these cells we sought to determine the phenotypic characteristics of cTfh and lymph node Tfh in blood and lymph node from tissues samples acquired simultaneously after HIV Envelope immunogen injection. Using sequential gating, Tfh were defined as live CD3+CD4+CD19-CD45RA-CXCR5+PD-1+ cells and cTfh as CD3+CD4+CD19-CD45RA-CXCR5+ cells (**Figure 4A**). Tfh were relatively frequent in LNC and rare or absent in PBMC, median (IQR) 1.0% (0.7-1.3%) vs 0.14% (0.09-0.17%), (p<0.001) (**Figure 4B**). By contrast, there was no difference in the frequency of cTfh in LNC and in PBMC median (IQR) 4.9% (3.4-6.6%) vs median (IQR) 7.8% (5.1-9.4%) (**Figure 4B**).

CD69 is a marker of T cell activation in peripheral blood and constitutively expressed on GC Tfh (19–21). To confirm our initial findings in Tfh and cTfh, separate immunophenotyping experiments were performed that included CD69 as a marker, from 4 participants (where cell numbers allowed). Data from paired LNC and PBMC, that had been rested overnight in cell culture medium from four participants were analyzed. Unsupervised clustering analysis was conducted on the concatenated data of CD4+ T cells from the paired left and right FNA and PBMC (**Figure 4C** and **Figure S2**). Expression of the immunophenotyping markers on the concatenated data was compared (**Figure 4D**). To dissect the immunophenotype of clusters exclusive to LNC or PBMC, the data were analyzed using FlowSOM establishing twenty populations (**Figure 4E**). Five populations were exclusive to LNC, and one population was exclusive to PBMC (**Figure 4F**). CD4+ T cell populations 3,4,5 and 6 highly expressed CXCR5 and all except population 4 were exclusive to LNC while population 10, which was exclusive to PBMC highly expressed PD-1 and CD95 (**Figure 4F** and **Table S2**).

Analysis of the four CD4+ T cell populations highly expressing CXCR5 (populations 3,4,5 and 6) confirmed population 4 to be distributed between blood and lymph node and populations 3, 5 and 6 to be present exclusively in lymph node and to express CD69 (**Figure 4G** and **Table S2**). The median fluorescence intensity (MFI) of the expression of CD69 on CD4+CD45RA-CXCR5+ Tfh cells was significantly higher among CD4+CD45RA-CXCR5+ Tfh cells in LNC than PBMC (p<0.0001). The MFI of PD-1 expression was highest on Tfh in LNC, lower on cTfh in PBMC (p<0.01) and significantly lower on CD4+ naïve T cells in PBMC (p<0.001) (**Figure 4H**). There was a dichotomous distribution of expression of CD69 (**Figure 4H**). These experiments established CD69, as highly expressed on Tfh in the lymph node but not on cTfh in the blood. A subset of CD4+ T cells in LNC (flow SOM population 6) were CD45RA-CCR7-CD28+PD-1^hi^CXCR5^hi^CD69^hi^CD95+CD137+CD154+IRF4+, a phenotype consistent with activated GC Tfh (**Table S2**).

### Induction of HIV-specific CD4+ T cells in lymph node

**Figure 5:**
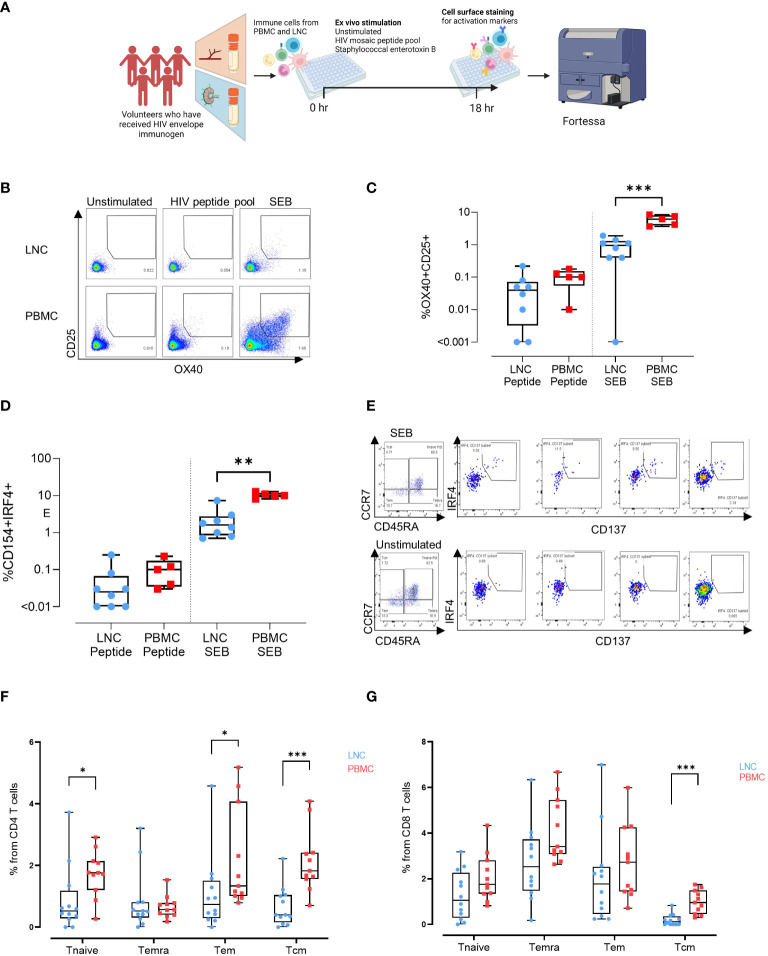
Induction of HIV-specific CD4+ T cells in lymph node and blunting of the superantigen response compared with the blood. **(A)** Stimulation experiments: Activation induced marker assay **(B)** Representative gating strategy for activation induced marker (AIM) assay. Cells were left unstimulated (left panel) or stimulated for 18 hours with a peptide pool covering the full length of the HIV Mosaic envelope trimers used in the immunogen injection (middle panel) or with staphylococcal enterotoxin B (right panel). Expression of CD25 and OX40 in CD4 T cells in LNC (top row) and PBMC (bottom row) is shown. **(C)** Box and whisker plot showing all points, lower quartile, median and upper quartile values for the LNC and PBMC stimulated with HIV MOSAIC peptide pool (left) or SEB (right) in the activation induced marker assay. Frequency of OX40+CD25+ cells in CD4+ T cells is shown **(D)** Box and whisker plot showing all points, lower quartile, median and upper quartile values for the LNC and PBMC stimulated with HIV MOSAIC peptide pool (left) or SEB (right) in the activation induced marker assay. Frequency of CD154+IRF4+ cells in CD4+ T cells is shown **(E)** Representative gating strategy for stimulation of LNC using SEB. CD8+ T cells are shown with panels from left to right showing Temra, Tcm, Tem and Tnaive subsets. **(F)** Comparison of activation status of T cell memory subpopulation in PBMC and LNC after stimulation with staphylococcal enterotoxin B PBMC and LNC were cultured with SEB for 18hrs in the IRF4 assay; n=6 for right FNA, n=6 for left FNA and n=11 for PBMC. Background signals were subtracted as follows: the signal in the antigen-stimulation minus the signal in the unstimulated condition. Frequencies shown are the percentage from the parent (CD4 or CD8) population. Frequencies of CD154+IRF4+ among CD4+T cells that are naïve (CD45RA+CCR7+), Temra (CD45RA+CCR7-), Tem (CD45RA-CCR7-) and Tcm (CD45RA-CCR7+) in LNC (blue circles), and from PBMC (red squares). **(G)** Frequency of CD137+ IRF4+ memory subpopulation in CD8+ T cells from the LNC (blue circles) and from PBMC (red squares). The results of Mann-Whitney U tests are shown with *p<0.05, **p<0.01, ***p<0.001.

To determine whether exposure to HIV Mosaic by injection induced CD4+ HIV-specific T cells in the lymph node we conducted *ex vivo* stimulation experiments. PBMC from n=5 participants and LNC from the same n=5 participants, were stimulated in an activation induced marker (AIM) assay using SEB and the HIV Mosaic peptide pool spanning the full length of the Env proteins used in the injection (**Figure 5A**). Events were gated based on time, and then light scatter properties, for singlets, live cells, CD4+ cells and then for expression of CD69 and CD3 (**Figure S3**). Activation of CD4+ T cells was marked by dual expression of CD25 and OX40 as previously described (**Figure 5B**) (19–21). Responders were classified as those with a frequency of CD25+OX40+ cells of 0.01% or above after normalization by subtracting the background response. Using this threshold, 6/8 LNC and 5/5 PBMC samples responded to the HIV immunogens and 7/8 LNC and 5/5 PBMC responded to the superantigen SEB. The frequency of responsive cells to the EAVI Mosaic peptide pool was equivalent in PBMC and LNC. Interestingly, the frequency of responses to SEB was significantly higher in PBMC than in LNC (p<0.001) (**Figure 5C**). Cells expressing CD154/CD40L were measured in the same assay (**Figure S4**); 5/8 LNC and 5/5 PBMC responded to the HIV immunogens and 8/8 LNC and 5/5 PBMC responded to the superantigen SEB. The frequency of responding cells to SEB in CD4+ T cells was similarly higher in PBMC (p<0.01) (**Figure 5D**).

IRF4 has a role in lymph node Tfh function and GC formation (in mice) (22). It is essential for the CD8+ T cell response to antigen through protection from cell death (in mice) and expression is dependent on TCR affinity (in humans) (23, 24). To compare expression of IRF4, PBMC and LNC were cultured with SEB for 18 hours and examined for expression of IRF4 and concomitant expression of CD154/CD40L in CD4+ cells and CD137/4-1BB in CD8+ T cells (n=6 for right FNA, n=6 for left FNA and n=11 for PBMC) (**Figure 5E** and **Figure S5**). In CD4+ T cells, cells that simultaneously expressed CD154 and IRF4 were more frequent in naïve (CD45RA+CCR7+), and Tem (CD45RA-CCR7-) in PBMC than LNC (p<0.05 for both) and particularly in Tcm (CD45RA-CCR7+) (p<0.001) (**Figure 5F**). Expression of IRF4 and CD154 in CD4+ T cells was therefore more sensitive to stimulation with SEB in PBMC than in LNC. A similar trend was seen in CD8+ T cells expressing CD137 and IRF4, which were significantly more frequently expressed in CD8+ Tcm in PBMC than in LNC where expression was negligible (p<0.001) (**Figure 5G**). Pairwise comparison between activation status of different memory subpopulations after SEB stimulation for 18 hours between left and right FNA, for IRF4+CD154+CD4+ T cell memory subpopulation and IRF4+CD137+CD8+ T cell memory subpopulation showed no significant difference (**Figure S6**).

### Naive CD8+ T cells in the lymph node are heterogenous

**Figure 6:**
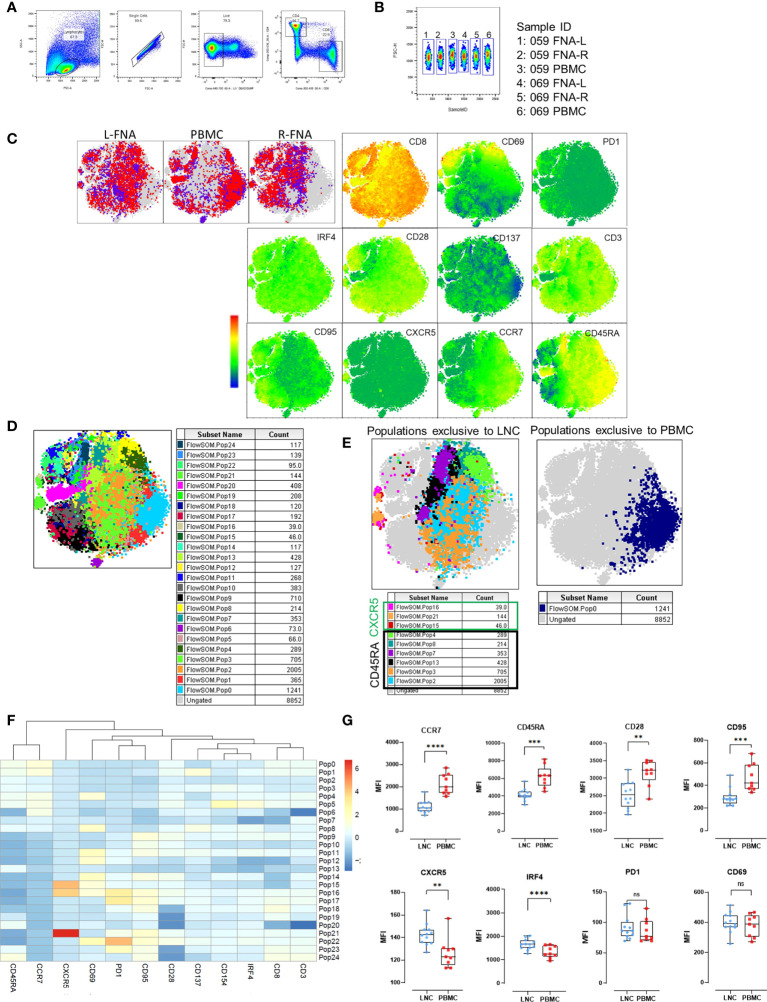
Phenotypic distinction of naïve-type CD8+ T cells in the lymph node compared with the blood. **(A)** Pairwise analysis of CD8+ T cells from down sampled data from LNC and PBMC (1531 events from each participant) was conducted (n=2) PBMC and LNC were cultured in media for 18 hours in the IRF4 assay. **(B)** Samples were identified using sample ID **(C)** t-SNE was run on these samples with perplexity of 30 Data were gated to identify the samples for L-FNA, PBMC and R-FNA using SampleID. Dots represent cells and color: purple and red represent the different participants and grey dots represent the ungated cells (cells that are absent in the gated subset). Expression of different markers is represented in the adjacent heatmap statistic plot. **(D)** 25 meta clusters detected by FlowSOM **(E)** Different subtypes of cells were identified by FlowSOM and plotted as subpopulations exclusive to LNC or PBMC using manual overlay. **(F)** Population heatmap showing relative expression of markers on the 25 metaclusters for FlowSOM populations are shown. **(G)** The median fluorescence intensity of different markers expressed on CD8+ T naive cells were compared by means of two-dimensional analysis. (n=12 LNC left and right and n=9 PBMC). *p<0.05, **p<0.01, ***p<0.001, ****p<0.0001.

To examine differences in CD8+ T cells observed in the two tissues, unsupervised pairwise analysis of CD8+ T cells from data from LNC and PBMC was conducted (n=2, where there were sufficient CD8+ T cell events from both left and right lymph nodes and PBMC for each participant). Equal numbers of events of CD8+ T cells (1531) were down-sampled from the data and t-SNE analysis conducted on the concatenated data. Samples from different tissues were compared using sample ID (**Figures 6A–C**). Several CD8+ T cell subtypes were abundant in LNC and absent in PBMC (**Figure 6C** and **Table S2**). FlowSOM analysis of the concatenated CD8+ T cell data (**Figure 6D**) generated 25 populations. A single cluster of CD8+ T cells with a naïve phenotype was exclusive to PBMC (population 0), six clusters were exclusive to LNC (Pop 2,3,4,8,7,13), and there were three rare CXCR5+CD8+ T cell subsets only in LNC (**Figure 6E, F**). To confirm if these phenotypic differences amongst CD8+ T naïve cells were present in all samples analyzed, subsequently conventional pairwise 2-dimensional analysis in unstimulated CD8+ T cells from PBMC (n=9) and left and right LNC (n= 12) was done. In CD8+ naïve T cells, CCR7, CD45RA and CD95, (measured by median fluorescence intensity) were most highly expressed in PBMC compared with LNC (p<0.0001 and p<0.001) and similarly CD28 (p<0.01). Conversely, CXCR5 and IRF4 were both more highly expressed in LNC than PBMC (p<0.01 and p<0.0001). PD-1, CD69 and CD137 (not shown) were not differently expressed on these CD8+ T cells between blood and lymph node (**Figure 6G**).

## Discussion

These data show that axillary human lymph nodes are enriched with LNC-associated CD4+ T cells, CD8+ T cells and B cells after intramuscular injection with HIV Env antigens. These LNC enriched cell types include CD4+ T cells specific for HIV Mosaic after an injection, in volunteers who had previously tested negative for HIV infection, and before the antibody response is detectable in the blood. The timing of these responses was similar to those in response to a vaccine of similar design in NHP (2). Given these cells make the initial response to antigen challenge by injection, they are likely critical for determining the outcome of the injection schedule and the breadth and potency of the immune response against HIV.

Several differences in adaptive immune cells and in antigen presenting cells known to be important in the vaccine reaction were evident between blood and lymph node. GC-like Tfh were restricted to the lymph node and expressed PD-1 and CD69. Conversely, CXCR5+PD-1-CD69- cTfh, were abundant in both tissues, similar to those previously described in the circulation and could belong to a subset of resting memory T cells (10, 25). In B cell subsets, plasmablasts were enriched in PBMC, consistent with their role in antibody secretion, whereas memory B cells were evident in both tissues. Our data raise the possibility that CD69+PD-1+ Tfh are a tissue-resident human lymph node population, whereas resting memory CD4+CXCR5+ T cells circulate between blood and lymph node. On deep immunophenotyping, LNC Tfh were CXCR5^hi^CD69^hi^ and expressed varying levels of PD-1, CD154 and IRF4. The PD-1^hi^ Tfh LNC co-expressed markers of activation consistent with Tfh gaining GC function, and this variable pattern of expression is resonant of the proposed cycling in and out of GC function during Tfh memory development (26). Simultaneous longitudinal analysis of blood and lymph node Tfh cell subsets in response to vaccination will further elucidate whether the CD69+PD-1+ Tfh we observed have other phenotypic and functional features of GC Tfh and how these are related to circulating Tfh.

CD14+ monocytes are an immature form of antigen presenting cell that is common in the blood, and these were virtually absent from LNC. As evidence of freedom from contamination with blood, this was an indicator of sample quality, like other studies (27, 28). Given the role of CD14+ monocytes in antigen presentation, this may have functional significance. Compared with PBMC, there were differences in the LNC responses to the superantigen SEB but not to the HIV Mosaic peptides. SEB binds and stabilizes the MHC Class II-T cell receptor complex formed by antigen presenting cells with T cells. The interaction bypasses the antigen-specific peptide binding site on the TCR making it a common immunological tool for T cell stimulation (29). Given that LNC are deficient in CD14+ monocytes, there may be a relative paucity of MHC class II molecules. This raises the question as to which LNC perform antigen presentation in antigen specific *ex vivo* assays and how this reflects *in vivo* function. MHC II expressing LNC B cells interacting with the TCR could deliver this function for B cell antigens, but the efficiency of B cells to do this presentation function for SEB is not clear and may be different from other antigen presenting cells (30).

Other differences were evident between blood and lymph node amongst adaptive immune cells, particularly T memory cells. LNC were abundant in CD4+ T cells, and CD8+ T cells less frequent than is usual in PBMC; a CD4:CD8 ratio three times higher than is typical in the blood was a distinguishing feature of LNC (31). LNC CD8+ T cells tended to have a naïve phenotype but were more heterogenous than naïve CD8+ T cells in the circulation. These CD8+ T cells in LNC had higher constitutive expression of IRF4 and CXCR5 and lower expression of CD28, CD95, CCR7 and CD45RA than their circulating counterparts. Populations exclusive to PBMC included CD4+ T cells that were expressors of CD95 (Fas) and PD-1, markers of apoptosis and exhaustion, a population that is described in peripheral blood of COVID-19 patients (32).

Axillary lymph node fine needle aspiration under ultrasound guidance was well-tolerated. The procedure was feasible with no specialist environment or equipment beyond those provided in a UK NHS clinical research setting. It was conducted at the bedside with no requirement for exposure to ionizing radiation (33).Our study had several limitations; it was performed during the COVID-19 pandemic with a necessarily pragmatic approach to the timing of study visits. It was not always possible to control for lymphatic stimulation by immunogens other than those used in the EAVI2020_01 study including COVID-19 vaccination. We demonstrated binding antibody responses to HIV; testing whether these antibodies have the properties of bNAbs is the subject of future studies. These findings indicate compartmental division of blood and lymph node underlying a tissue-specific response to vaccination. Although previous FNA studies are scarce, elegant observations on B cells by Ellebedy and colleagues demonstrating differences in the kinetics and mutational burden of circulating and GC B cells after vaccination support this hypothesis (27, 28, 34). The pivotal nature of engagement of GC Tfh in the serological reaction to SARS-CoV-2 vaccination has been shown in one study of drug-induced immunocompromise (35). However, data on human lymph node T cells are remarkably few and are confined to a few vaccines including studies of mRNA COVID-19 and tuberculin PPD vaccines (4, 35). The distinguishing features which we demonstrate in human LNC, which are unlike those of the cells in the blood raise the possibility for enhanced understanding of the immune system in vaccine studies. The development of a successful HIV vaccine; its antigen, adjuvant, formulation, dosing, and scheduling is dependent on inducing a robust and sustained GC reaction to yield bNAbs. This will be more efficiently achieved through adoption of the direct study of adaptive cellular evolution in lymph nodes during experimental immunization.

## Data availability statement

The original contributions presented in the study are included in the article/**Supplementary Materials** further inquiries can be directed to the corresponding author.

## Ethics statement

The studies involving human participants were reviewed and approved by The EAVI2020_01 study (NCT04046978) was approved by London – Fulham Research Ethics Committee (18/LO/2196). The patients/participants provided their written informed consent to participate in this study.

## Author contributions

Conceptualization: NL, RS, KP. Methodology: SD, CK, HC, NL, AL. Investigation: SD, CK, LM. Visualization: SD, CK. Formal analysis: SD, CK, HC, EG, LM, KP. Resources: HC, EG, MT, SC, AL, RS, RS, KP. Data curation HC, EG, MT. Funding acquisition: BA, RS, RS, KP. Project administration: HC, TC, RS, KP. Supervision: BA, KP. Writing – original draft: SD, KP. Writing – review & editing: SD, CK, HC, BA, RS, KP. All authors contributed to the article and approved the submitted version.

## Funding

European Union’s Horizon 2020 research and innovation programme grant 681137 (RS). The sole responsibility for the content of this project lies with the authors. It does not necessarily reflect the opinion of the European Union. The European Commission is not responsible for any use that may be made of the information contained therein. St Mary’s Development Trust fellowship (KP); European Union’s Horizon 2020 research and innovation programme grant 764698 (QUANTII) Wellcome Trust (WT) Investigator (103865Z/14/Z), Medical Research Council (MC) (J007439, G1001052), European Union Seventh Framework Programme (FP7/2007–2013) grant 317040 (QuanTI), Leukemia and Lymphoma Research (15012) (BA). Infrastructure support was provided by the NIHR Imperial Biomedical Research Centre and the NIHR Imperial Clinical Research Facility. The views expressed are those of the author(s) and not necessarily those of the NHS, the NIHR or the Department of Health and Social Care.

## Acknowledgments

The authors would like to thank the participants in this study and the staff of the NIHR Imperial Clinical Research Facility and wish to acknowledge the support of the St Mary’s Flow Cytometry Core Facility at Imperial. The authors would like to thank Dr John Tregoning for critical appraisal of the manuscript.

## Conflict of interest

KP is chief, principal or co-investigator for vaccine clinical trials and experimental medicine studies (NCT05007275, NCT04753892, EudraCT 2020-001646-20, NCT04400838, NCT04324606, EudraCT 2017-004610-26, NCT03970993, NCT03816137), is a member of the data safety monitoring board for NCT05249829, has received a fee for speaking from Seqirus and Sanofi Pasteur, and has research funding from the Chan Zuckerberg Initiative, the MRC/UKRI, the Vaccine Task Force, and NIHR Imperial BRC outside the submitted work.

The remaining authors declare that the research was conducted in the absence of any commercial or financial relationships that could be construed as a potential conflict of interest.

## Publisher’s note

All claims expressed in this article are solely those of the authors and do not necessarily represent those of their affiliated organizations, or those of the publisher, the editors and the reviewers. Any product that may be evaluated in this article, or claim that may be made by its manufacturer, is not guaranteed or endorsed by the publisher.
