# Supplementary material for: Comparison of blood and lymph node cells after intramuscular injection with HIV envelope immunogens
Source: Front Immunol. 2022 Oct 5;13:991509. doi: 10.3389/fimmu.2022.991509 (PMC9579690; doi:10.3389/fimmu.2022.991509)
Supplement: Supplementary file 1 [file DataSheet_1.docx]

**Comparison of blood and lymph node cells after intramuscular injection with HIV envelope immunogens**

**Supplementary Materials and Methods**

**Inclusion and exclusion criteria**

All participants had previously been judged to have met inclusion and exclusion criteria for the parent study with a negative result in a 4th generation HIV antigen/antibody test. Participants were excluded from the FNA study if they had taken blood thinning medication likely to induce bruising prior to biopsy, had signs of local infection, had pain or swelling at any sites of potential lymph node sampling, had a history of allergy to local anaesthetic, were unwilling or unable to provide written, informed consent for the procedure, or during medical history taken revealed any other medical reason, which the investigator deemed significant to warrant exclusion from the FNA sub-study.

**FNA sampling procedure and arrangements**

Following eligibility assessment, a skilled medical practitioner carried out the FNA using clinical facilities at Imperial College Healthcare NHS Trust, London, UK with support from a trained clinical research nurse. The FNA was conducted using standard aseptic technique. During the procedure, the ipsilateral and contralateral lymph nodes in the axilla were located by examination and then under ultrasound guidance. A Toshiba Aplio i300 ultrasound machine was used to convey standard greyscale ultrasound. The area was cleaned using 0.5% chlorhexidine and 1-5 mL 1% lidocaine infiltrated into the surrounding tissue. A sterile 21G needle and 5ml syringe was used to aspirate material from lymph nodes on each side using three passes under ultrasound guidance. Each visit lasted approximately 90 minutes.

**Expected Adverse Events and Grading**

Expected adverse events following lymph node biopsy included pain or tenderness at the sample site. Bleeding and bruising were expected to be mild. Adverse events related to the procedure were solicited immediately afterwards and at the next study visits. Participants were provided with information regarding expected adverse events in a participant information leaflet.

**Reimbursement**

Participants in the study were offered reimbursement of £100 for each biopsy visit for time, inconvenience, and travel payable at the end of study participation.

**Stopping/holding criteria**

For any participant, FNA could be discontinued at the visit if any issues with tolerability arose, and participants could choose to withdraw from sampling at any time without affecting their medical care or further participation in the overall EAVI2020_01 study. Lymph node sampling for all participants could be paused in the case of any serious adverse event arising in any participant that was deemed related to the procedure.

**Sample collection**

500 mL sterile filtered R10 media; Roswell Park Memorial Institute medium 1640 (RPMI) (Gibco), 10% heat inactivated fetal bovine serum (Sigma), Penicillin/Streptomycin at a working concentration of 100 U/mL/100 µg/mL) (Gibco) and L-glutamine (2mM) (Gibco) was prepared using standard procedure in an MSc cabinet and stored at 2-8^o^C when not in use.

Specimen pots were prepared up to one week in advance, with two tubes required per sampling timepoint, one for each axilla. 15 mL Falcon tubes were prepared with 10-15 mL of sterile filtered R10 and labelled with expiry date and study number, outside of tube cleaned with 70% alcohol solution or similar such as 70% IMS and kept refrigerated at 2-8^o^C until required.

Each FNA sample was transferred from needle into labelled specimen pots, one for each side and flushed with R10 media to dislodge cells. 42 mL of whole blood was collected into 1 x 6 mL serum tube and 6 x 6 mL sodium heparin tube using standard clinical phlebotomy technique. Samples were placed in Air Sea containers and transfer samples by courier (on ice) to the clinical trial laboratories (St Mary’s Campus, Imperial College London, London, UK), and processed upon receipt.

**Sample processing**

Peripheral blood mononuclear cells and serum samples were prepared as per standard established laboratory protocols. Briefly LNC were processed by lysing red blood cells using 5mL red blood cell lysis buffer (Gibco, Cat: A10492-01) centrifuged at 400 g for 10 mins and washed twice with cell wash buffer (PBS with 2% FBS and 2mM EDTA) before the final volume was adjusted to 5 mL in R10. Cells were counted using a cell counter (Beckman Coulter ViCell) or by detecting cell viability trypan blue exclusion and the cell numbers and viability from each sample recorded. Samples were either used immediately in experimental assays or frozen in 10% DMSO in HI-FBS according to standard viable cryopreservation protocols.

**
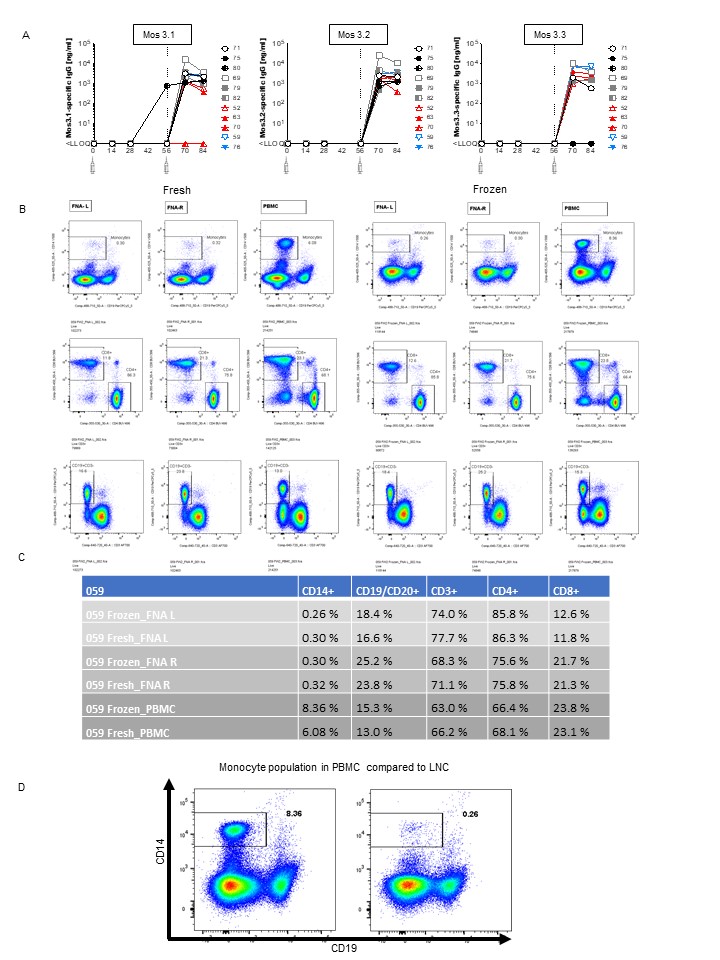
**

**Figure S1. Immune responses, gating strategy and comparison of freshly prepared LNC with viably cryopreserved LNC. (A)** HIV-specific IgG (ng/mL) against Mos 3.1 (left panel), Mos 3.2 (middle panel) and Mos3.3 (right panel) from individual participants in group 1 (black circles), group 2 (grey squares), group 3 (red triangles) and group 4 (blue inverted triangles). **(B)** LNC were stained with EAVI202001_FNAcocktail 1 on the day of sample processing. The remainder were viably cryopreserved in 10% DMSO/FBS, stored at -150^o^C for 1 week, then raised, thawed, and stained and acquired in the same way

**(C)** Table comparing major frequencies of major subsets amongst LNC and PBMC

**(D)** Frequency of CD14+ monocytes amongst PBMC (left panel) and LNC (right panel)

**
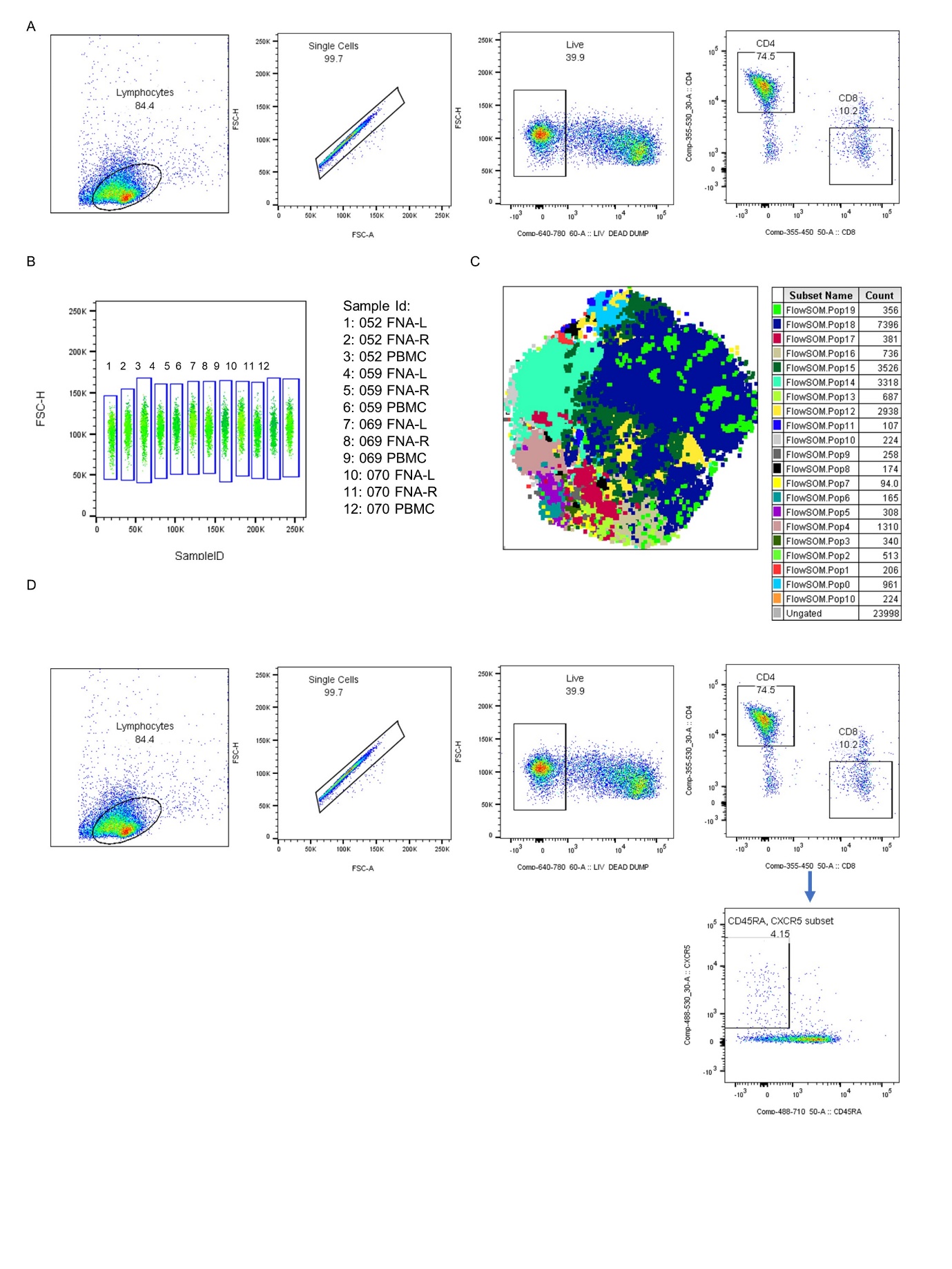
Figure S2. Gating strategy for CD4+ T cells for tSNE and FlowSOM analysis**

**(A)** Representative dot plots from one lymph node sample

**(B)** Equal number of events were downsampled from CD4+ T cells from all the participants and concatenated. To identify the samples, cells were gated on SampleID

**(C)** t-SNE plots representing the 20 meta clusters detected by FlowSOM.

**(D)** Gating strategy for CD4+CXCR5+CD45RA- Tfh cells from one representative sample of lymph node sample to compare the MFI of CD69 and PD1.


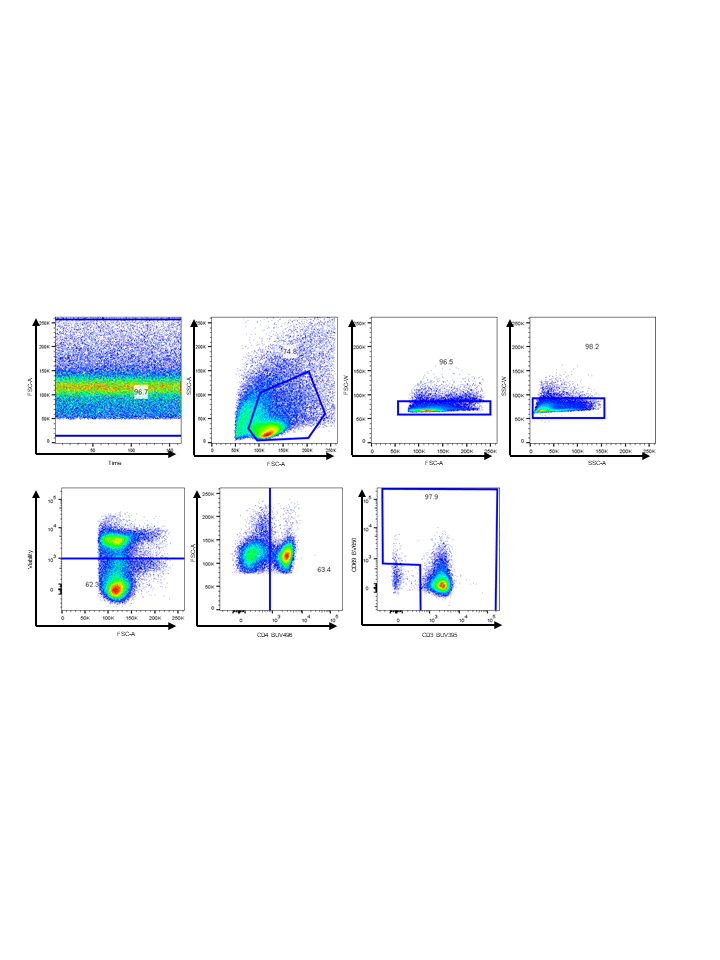
**Figure S3. Gating strategy for activation induced marker assay;** PBMC are shown. Representative flow cytometry for AIM assay ​


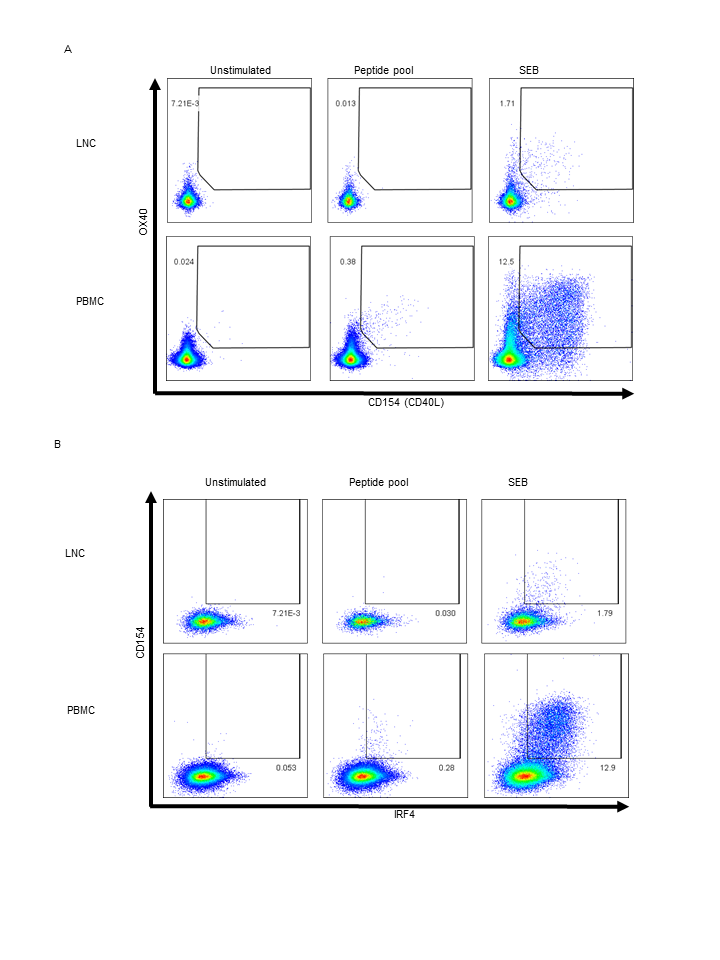


**Figure S4. Activation markers in activation induced marker assay**

(A) Comparing unstimulated (left panel), HIV peptide pool stimulated (middle panel) and SEB stimulated cells (right panel) from LNC (upper row) and PBMC (lower row).

(B) Activation markers in IRF4 assay comparing unstimulated (left panel), HIV peptide pool stimulated (middle panel) and SEB stimulated cells (right panel) from LNC (upper row) and PBMC (lower row).


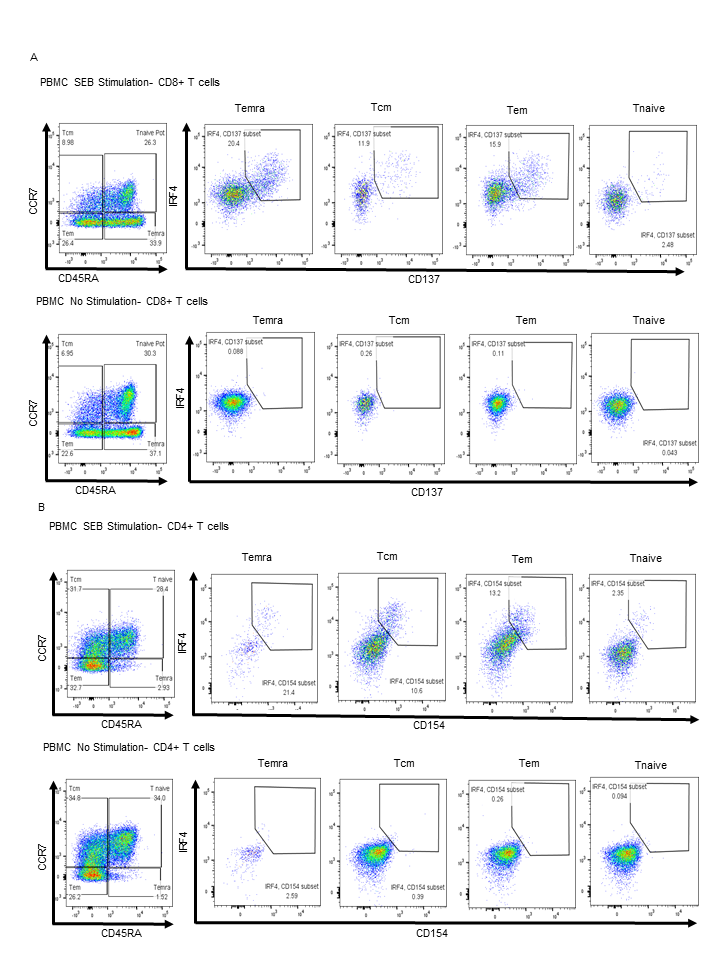
**Figure S5** **Gating strategy for IRF4+CD137+ CD8+ T cells and IRF4+CD154+ CD4+ T cells** (A) for PBMC gated on CD8+ T cells: SEB stimulated and unstimulated

(B) for PBMC for gated on CD4+ T cells: SEB stimulated and unstimulated


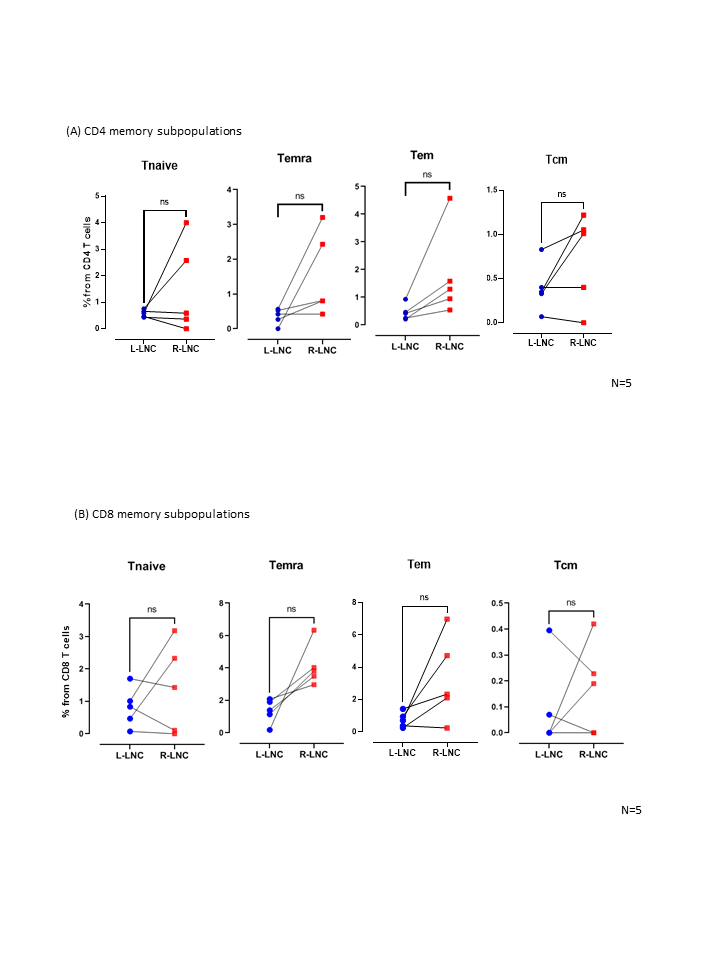


**Figure S6 Pairwise comparison between activation status of different memory subpopulations between Left and Right FNA**. Cells were cultured with SEB stimulation for 18hrs and analysed for the IRF4+CD154+CD4+ T cell memory subpopulation IRF4+CD137+CD8+ T cell memory subpopulation. Results show Wilcoxon match paired rank test (ns = non-significant).

**Table S1 Cellular yields from FNA and experiments performed**

| **Participant number** | **Date** | **Left axilla count (x10^6^)** | **Left axilla viability (%)** | **Right axilla count (x10^6^)** | **Right axilla viability (%)** | **Pheno-typing** | **AIM assay** | **IRF4 assay** |
| --- | --- | --- | --- | --- | --- | --- | --- | --- |
| 070 | 10 June 2021 | 5.4 | 97.1 | 2.8 | 91.9 | y | y | y |
| 079 | 10 June 2021 | 0.45 | NR | 0.16 | 97.1 | n | n | y |
| 069 | 10 June 2021 | 7.25 | 92.6 | 2.88 | 89.7 | y | y | y |
| 052 | 24 June 2021 | 5.7 | 98.6 | 0.99 | 95.8 | y | y | y |
| 082 | 24 June 2021 | 0.08 | 50.0 | 0.16 | 44.4 | n | n | y* |
| 071 | 01 July 2021 | 0.16 | 57.1 | 0.45 | 52.4 | n | n | y* |
| 075 | 08 July 2021 | 0.35 | 69.2 | 1.3 | 61.5 | n | n | y |
| 076 | 15 July 2021 | 1.40 | 77.3 | 0.25 | 42.9 | y | n | y |
| 080 | 22 July 2021 | 0.41 | 71.4 | 0.49 | 100 | n | n | y* |
| 063 | 29 July 2021 | 6.25 | 96.7 | 10.5 | 90.6 | y | y | y |
| 059 | 05August 2021 | 3.99 | 90.7 | 2.22 | 98.2 | y | y | y |

* Insufficient cells for analysis: 071, 080 and 082 were only run on IRF4 assay and FNA samples had insufficient cells for analysis therefore data were excluded

| **LNC or PBMC** | **FlowSOM population** | **Memory phenotype or lymphatic tissue migration** | **Costimulatory, chemokine receptor, tissue residency or activation and cell signalling** | **Activation and transcription factors** |
| --- | --- | --- | --- | --- |
| **CD3+CD4+** | | | | |
| **LNC** | 2 | CD45RA-CCR7- | CD28-PD-1+CXCR5-**CD69^hi^**CD95+ | CD137+CD154-IRF4- |
|  | 3 | CD45RA-CCR7- | CD28-PD-1+**CXCR5^hi^CD69^hi^**CD95+ | CD137+CD154-IRF4- |
|  | 5 | CD45RA-CCR7- | CD28+PD-1+**CXCR5^hi^CD69^hi^**CD95+ | CD137+CD154+IRF4+ |
|  | 6 | CD45RA-CCR7- | CD28+**PD-1^hi^CXCR5^hi^CD69^hi^**CD95+ | CD137+CD154+IRF4+ |
|  | 12 | CD45RA+CCR7- | CD28^lo^PD-1-CXCR5-CD69+CD95- | CD137-CD154-IRF4^lo^ |
| **PBMC** | 10 | CD45RA-CCR7- | CD28+**PD-1^hi^** CXCR5-CD69-**CD95^hi^** | CD137+CD154+IRF4+ |
| **CD3+CD4+CXCR5+** | | | |  |
| **LNC** | 3 | CD45RA-CCR7- | CD28-PD-1+ **CXCR5^hi^CD69^hi^**CD95+ | CD137+CD154-IRF4- |
|  | 5 | CD45RA-CCR7- | CD28+ PD-1+ **CXCR5^hi^CD69^hi^**CD95+ | CD137+CD154+IRF4+ |
|  | 6 | CD45RA-CCR7- | CD28+ **PD-1^hi^CXCR5^hi^CD69^hi^**CD95+ | CD137+CD154+IRF4+ |
| **PBMC and LNC** | 4 | CD45RA-CCR7+ | CD28+ PD-1-**CXCR5^hi^**CD69-CD95+ | CD137+CD154+IRF4+ |
| **CD3+CD8+** | | | |  |
| **LNC** | 2 | CD45RA+CCR7+ | CXCR5-CD69-PD-1-CD95-CD28- | CD137-CD154-IRF4- |
|  | 3 | CD45RA+CCR7+ | CXCR5-CD69-PD-1-CD95-CD28- | CD137-CD154+IRF4- |
|  | 4 | CD45RA+CCR7+ | CXCR5-CD69+PD-1-CD95-CD28+ | CD137-CD154-IRF4- |
|  | 7 | CD45RA+CCR7+ | CXCR5-CD69-PD-1-CD95-CD28- | CD137-CD154-IRF4^lo^ |
|  | 8 | CD45RA+CCR7+ | CXCR5-CD69+PD-1-CD95-CD28- | CD137+CD154-IRF4- |
|  | 13 | CD45RA+CCR7- | CXCR5-CD69-PD-1-CD95-CD28- | CD137-CD154-IRF4^lo^ |
| **PBMC** | 0 | CD45RA+CCR7+ | CXCR5-CD69-PD-1-CD95-CD28+ | CD137-CD154-IRF4- |
| **CD3+CD8+CXCR5+** | | | | |
| **LNC** | 15 | CD45RA-CCR7- | **CXCR5^hi^** CD69+PD-1-CD95-CD28- | CD137-CD154-IRF4- |
|  | 16 | CD45RA-CCR7- | **CXCR5^hi^**CD69+**PD-1^hi^** CD95+CD28+ | CD137+CD154+IRF4+ |
|  | 21 | CD45RA-CCR7- | **CXCR5^hi^**CD69-PD-1+CD95+CD28+ | CD137-CD154-IRF4- |

**Table S2 CD4+ and CD8+ T cell subsets identified by visual discrimination using FlowSOM analyses**
